# 2D DIGE proteomic analysis reveals fasting‐induced protein remodeling through organ‐specific transcription factor(s) in mice

**DOI:** 10.1002/2211-5463.12497

**Published:** 2018-08-13

**Authors:** Shotaro Kamata, Junya Yamamoto, Haruka Ohtani, Yuka Tosaka, Sayumi Yoshikawa, Noriyuki Akahoshi, Isao Ishii

**Affiliations:** ^1^ Laboratory of Health Chemistry Showa Pharmaceutical University Tokyo Japan; ^2^ Laboratory of Biochemistry Keio University School of Pharmaceutical Sciences Tokyo Japan

**Keywords:** 2D DIGE, fasting, PPARα, proteomics, TP53, transcriptional regulation

## Abstract

Overnight fasting is a routine procedure before surgery in clinical settings. Intermittent fasting is the most common diet/fitness trend implemented for weight loss and the treatment of lifestyle‐related diseases. In either setting, the effects not directly related to parameters of interest, either beneficial or harmful, are often ignored. We previously demonstrated differential activation of cellular adaptive responses in 13 atrophied/nonatrophied organs of fasted mice by quantitative PCR analysis of gene expression. Here, we investigated 2‐day fasting‐induced protein remodeling in six major mouse organs (liver, kidney, thymus, spleen, brain, and testis) using two‐dimensional difference gel electrophoresis (2D DIGE) proteomics as an alternative means to examine systemic adaptive responses. Quantitative analysis of protein expression followed by protein identification using matrix‐assisted laser desorption ionization–time‐of‐flight mass spectrometry (MALDI‐TOFMS) revealed that the expression levels of 72, 26, and 14 proteins were significantly up‐ or downregulated in the highly atrophied liver, thymus, and spleen, respectively, and the expression levels of 32 proteins were up‐ or downregulated in the mildly atrophied kidney. Conversely, there were no significant protein expression changes in the nonatrophied organs, brain and testis. Upstream regulator analysis highlighted transcriptional regulation by peroxisome proliferator‐activated receptor alpha (PPARα) in the liver and kidney and by tumor protein/suppressor p53 (TP53) in the thymus, spleen, and liver. These results imply of the existence of both common and distinct adaptive responses between major mouse organs, which involve transcriptional regulation of specific protein expression upon short‐term fasting. Our data may be valuable in understanding systemic transcriptional regulation upon fasting in experimental animals.

**Abbreviations**
2D DIGE: two‐dimensional difference gel electrophoresis
DTT: dithiothreitol
GIP: gastric inhibitory polypeptide
IEF: isoelectric focusing
IPG: immobilized pH gradient
MALDI‐TOFMS: matrix‐assisted laser desorption ionization–time‐of‐flight mass spectrometry
PPARα: peroxisome proliferator‐activated receptor alpha
PPREs: peroxisome proliferator response elements
TP53: tumor protein/suppressor p53

Fasting has been practiced for millennia in religious ceremonies among Christians, Muslims (e.g., Ramadan), Buddhism, Jews, Hindus, and others, to reduce physical activities, resulting in a state of ‘quiescence’ like death. It has also been practiced in clinical situations to prevent obesity, hypertension, asthma, rheumatoid arthritis, and seizures 1, 2. Moreover, in current clinical settings and basic research using animals, overnight fasting is a routine procedure before surgical operations although its influences not directly related to organs of interest or investigated parameters are often ignored 3.

Maintaining adequate blood levels of glucose is prerequisite for energy metabolism in glucose‐requiring organs/cells including the brain, kidney, testis, and red blood cells. Upon food deprivation, declining blood glucose levels induce rapid secretion of glucagon and decreased release of insulin, thereby activating hepatic glycogenolysis although hepatic glycogen becomes quickly (~ 24 h) depleted. If fasting continues, peripheral organs switch as the primary energy source from glucose to fatty acids that are released from triacylglycerol droplets in adipose tissues. However, some organs/cells are unable to utilize fatty acids as an energy source, and thus, the liver produces ketone bodies from fatty acids so that such organs/cells can use them as a secondary energy source and save glucose. Meanwhile, gluconeogenesis from glucogenic amino acids of protein origin and ketogenesis from ketogenic amino acids takes place to maintain blood glucose and energy sources, respectively. Several lines of evidence suggest that all such biochemical adaptation to fasting is ‘transcriptionally regulated’ in the liver. The transcriptional factor/nuclear receptor proliferator peroxisome proliferator‐activated receptor alpha (PPARα) has been shown to primarily mediate adaptive responses to fasting in the liver 4, 5, 6, 7. In addition, tumor protein/suppressor p53 (TP53) has been shown recently to increase via posttranscriptional regulation in the liver upon fasting, thereby mediating amino acid catabolism and gluconeogenesis 8. However, such transcriptional regulation upon fasting has not been described in nonhepatic organs. Moreover, proteomic studies on the nonhepatic organs during fasting have been unexpectedly limited 9, 10 although the impacts of fasting are often apparent in the nonhepatic organs as we reported fasting‐induced cardioprotection in mice 11.

In this study, we investigated the possible sources of organ‐specific transcriptional regulation upon fasting using two‐dimensional difference gel electrophoresis (2D DIGE) proteomic approach. Although LC‐MS/MS became the mainstream for such proteomic analysis in recent decades, conventional 2D DIGE continues to be an important technology that enables rapid and direct visualization of thousands of proteins and their quantitative analyses 12, 13, 14, 15, 16, 17, 18. Here, we report novel transcriptional regulation in nonhepatic organs including kidney, thymus, and spleen upon fasting for 2 days.

## Materials and methods

### Animals

C57BL/6J mice were purchased from Japan SLC (Shizuoka, Japan). Eight‐week‐old male mice were group‐housed (4 mice per 470‐cm^2^ cage) in an air‐conditioned room (24 °C) kept on a 12‐h dark/light (8 pm–8 am) cycle, and allowed free access to water and a CE‐2 standard dry rodent diet (Clea Japan, Tokyo, Japan). In fasting experiments, mice were deprived of the diet for 1 or 2 days between 2 pm and 2 pm (hereinafter referred to as F1 and F2 mice, respectively). *Ad libitum*‐fed (AL) mice were analyzed as controls. After anesthetization by isoflurane inhalation, blood was collected through the heart and the liver, kidney, thymus, spleen, brain, and testis were quickly dissected out, snap‐frozen by liquid nitrogen, and stored at −80 °C. All animal procedures conformed to the Guide for the Care and Use of Laboratory Animals, 8^th^ Edition published by the US National Research Council, and were approved by the Animal Care Committees of Keio University (No. 09187‐[4–6]) or Showa Pharmaceutical University (No. P‐2016‐10).

### Serum biochemistry

Serum levels of glucose and ketone bodies were measured using a Drichem 7000i biochemistry analyzer (Fujifilm, Tokyo, Japan) and an AutoWako Total Ketone Bodies clinical assay kit (Wako, Osaka, Japan), respectively. Serum levels of adiponectin, insulin, C‐peptide 2, leptin, resistin, and gastric inhibitory polypeptide (GIP; also known as glucose‐dependent insulinotropic polypeptide 19) were measured using Multiplex Biomarker Immunoassays for Luminex xMAP technology (Millipore, Billerica, MA, USA); Catalog Nos. MADPNMAG‐70K‐01 for adiponectin; and MMHMAG‐44k for other hormones. Quantitative analyses were performed using Luminex xPONENT and MILLIPLEX Analyst 4.2 software.

### 2D DIGE

Each organ aliquot (50–100 mg) was homogenized (4100 r.p.m., 30 s × 3, 4 °C) in ice‐cold urea buffer (7 m urea, 2 m thiourea, 4% CHAPS, 65 mm dithiothreitol [DTT], 1 mm phenylmethylsulfonyl fluoride, and 1 mm sodium orthovanadate) using a Micro Smash MS‐100R Beads Cell Disrupter (Tomy, Tokyo, Japan) and 5‐mm‐diameter zirconia beads (Tomy). Homogenates were centrifuged at 16 000 ***g*** for 5 min at 4 °C, and then, the supernatants were centrifuged at 20 000 ***g*** for 25 min at 4 °C. Protein concentrations of the resultant supernatants were determined using a Bio‐Rad Protein Assay and bovine serum albumin as a standard. All reagents used in this study were of analytical grades from Wako (Tokyo, Japan) or Sigma‐Aldrich unless otherwise noted. 2D DIGE was performed as described previously 12, 13, 14. Twenty‐five μg of protein (adjusted to pH 8.5 by adding 40 mm Tris/HCl [pH 8.5]) was labeled with 200 pmol of CyDye (Cy2, Cy3, or Cy5 minimal dye fluor [GE Healthcare]) for 30 min at 4 °C in the dark. A pool, to be used for calibration between the gels, was generated from equal protein amounts of all eight samples (*n* = 4 each for AL and F2 mice). The reaction was stopped by adding 0.5 μL of 10 mm lysine. Labeled samples were mixed, and DTT and immobilized pH gradient (IPG) buffer (final 1% each) were added for 10 min at 4 °C in the dark. The samples were subjected to isoelectric focusing (IEF) in an Immobiline DryStrip (18 cm, pH 3–10 NL [nonlinear], GE Healthcare) that was rehydrated for 20 h in rehydration buffer (7 m urea, 2 m thiourea, 2% Triton X‐100, 13 mm DTT, 2.5 mm acetic acid, 1% IPG buffer, and 5 p.p.m. bromophenol blue) at 20 °C, using a CoolPhoreStar IPG‐IEF Type‐PX system (Anatech, Tokyo, Japan). Once IEF was completed, the strips were equilibrated for 30 min in equilibration buffer (50 mm Tris/HCl [pH 6.8], 6 m urea, 2% SDS, 30% [v/v] glycerol, 65 mm DTT, and 5 p.p.m. bromophenol blue), followed by in alkylating buffer (equilibration buffer with 4.5% iodoacetamide instead of DTT) for an additional 15 min. The strips were sealed on the top of 12.5% PAGE gels (140 × 140 × 1 mm; Perfect NT Gel S from DRC, Tokyo, Japan) using 0.5% low‐melting‐point agarose in Tris/glycine electrophoresis buffer. The second dimension of protein separation was performed at a constant 200 V using an ERICA‐*S* high‐speed electrophoresis system (DRC). A total of four gels (for the comparisons between AL (*n* = 4) and F2 (*n* = 4)) were scanned at once for Cy2/Cy3/Cy5 fluorescence using a Typhoon Trio image scanner (GE Healthcare), and obtained images were integratively analyzed using DeCyder 2D ver. 6.5 differential analysis software (GE Healthcare).

### Silver staining

For protein identification using MALDI‐TOFMS, each tissue homogenate sample (100–150 μg) was subjected to 2D PAGE (mentioned above) without CyDye labeling. To get better resolutions, some samples were separated on larger 2D systems using longer strips (24 cm, pH 3–10 NL [nonlinear]) and larger PAGE gels (257 × 200 × 1 mm; Perfect NT Gel W from DRC). After electrophoresis, the gel was stained using a Silver Stain MS Kit (Wako).

### MALDI‐TOFMS analysis of trypsin digests

Gel pieces were excised from silver‐stained gels, destained with a mixture of destaining solutions A and B (Wako), washed twice with deionized water and four times with 50 mm ammonium bicarbonate (NH_4_HCO_3_):acetonitrile (1 : 1), dehydrated once with acetonitrile, twice alternately rehydrated with 100 mm NH_4_HCO_3_ and dehydrated with acetonitrile, and dried by vacuum centrifugation. Protein samples in the gels were digested in 10 μL of trypsin solution (0.1 μg of Trypsin Gold, Mass Spectrometry Grade [Promega] and 0.01% ProteaseMAX Surfactant, Trypsin Enhancer [Promega] in 25 mm NH_4_HCO_3_) by incubating at 50 °C for 1 h. Trypsin digests were mixed with 3 μL of 2% trifluoroacetic acid, and 1 μL of samples was spotted onto a μFocus MALDI plate (900 μm, 384 circles, Hudson Surface Technology [Old Tappan, NJ, USA]) with an equal volume of matrix solution, containing 10 mm α‐cyano‐4‐hydroxycinnamic acid in 1% trifluoroacetic acid/50% acetonitrile. Positive ion mass spectra were obtained using an AXIMA‐CFR plus (Shimadzu, Kyoto, Japan) in a reflectron mode. MS spectra were acquired over a mass range of 700–4000 *m*/*z* and calibrated using Peptide calibration standards (~ 1000–3200 Da; Bruker Daltonics, Yokohama, Japan).

### Database search for protein identification/clarification and upstream regulator analysis

Proteins were identified by matching the peptide mass fingerprint with the Swiss‐Prot protein database using the MASCOT search engine (Matrix Science, http://www.matrixscience.com). Database searches were carried out using the following parameters: taxonomy, *Mus musculus*; enzyme, trypsin; and allowing 1 missed cleavage. Carbamidomethylation was selected as a fixed modification, and the oxidation of methionine was allowed as a variable. The peptide mass tolerance was set at 0.5 Da, and the significance threshold was set at *P* < 0.05 probability based values on Mowse Scores (≥ 55). Protein classification by its biological process involved and its molecular function was carried out using the PANTHER (Protein ANalysis THrough Evolutionary Relationships) clarification system (http://www.pantherdb.org/), which is supported by a research grant from the National Institute of General Medical Sciences [Grant GM081084] and maintained by the group led by Paul D. Thomas at the University of Southern California. Upstream regulator analysis was performed using Ingenuity Pathway Analysis (IPA) software (Qiagen).

### Statistical analysis

Data are expressed as mean ± SD (*n*: sample numbers). Statistical analysis was performed using one‐way ANOVA with Tukey's multiple comparison test with Prism ver. 5.0c software (GraphPad, La Jolla, CA, USA); *P* < 0.05 denoted a significant difference.

## Results

### Protein expression changes in the liver

**Figure 1:**
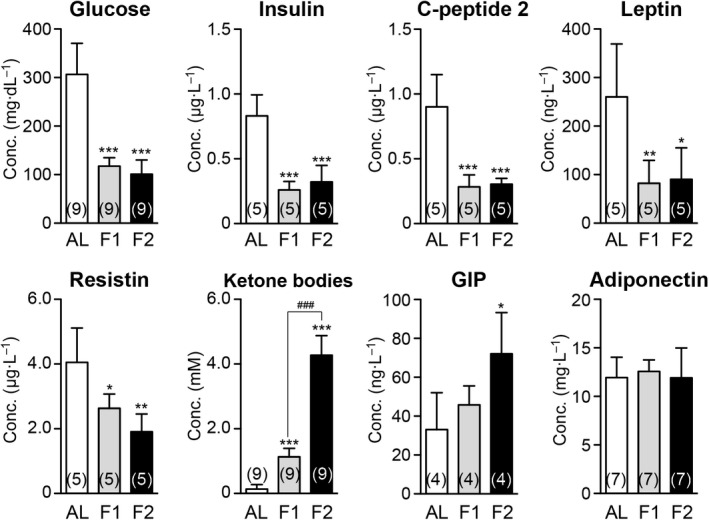
Impact of 1‐ or 2‐day fasting on serum biochemical parameters. Serum levels of glucose, insulin, C‐peptide 2, leptin, resistin, ketone bodies, gastric inhibitory polypeptide (GIP), and adiponectin were measured. AL,* ad libitum*‐fed (AL); F1, 1‐day fasted; F2, 2‐day fasted. Data are mean ± SD (*n*: sample numbers); significant changes in *P < 0.05, **P < 0.01, and ***P < 0.001 vs AL; ^###^ *P* < 0.001 vs F1 by one‐way ANOVA with Tukey's multiple comparison test.

We first estimated the duration required to obtain organ proteomic responses by fasting using serum biochemistry. One‐day (water‐only) fasting was sufficient to maintain minimal levels of glucose, insulin, C‐peptide 2, leptin, and resistin; the levels generally matched those in F2 mice, but, in contrast, the accumulation of ketone bodies and GIP was much more apparent in F2 mice (Fig. 1). Our exploratory 2D DIGE analyses did not find apparent alterations in hepatic protein expression in F1 mice (data not shown), and additional (e.g., 3‐day) fasting (that may cause acute > 25% body weight loss) was not allowed for ethical reasons in our university. Therefore, we investigated global protein expression in various mouse organs after 2‐day fasting.

**Figure 2:**
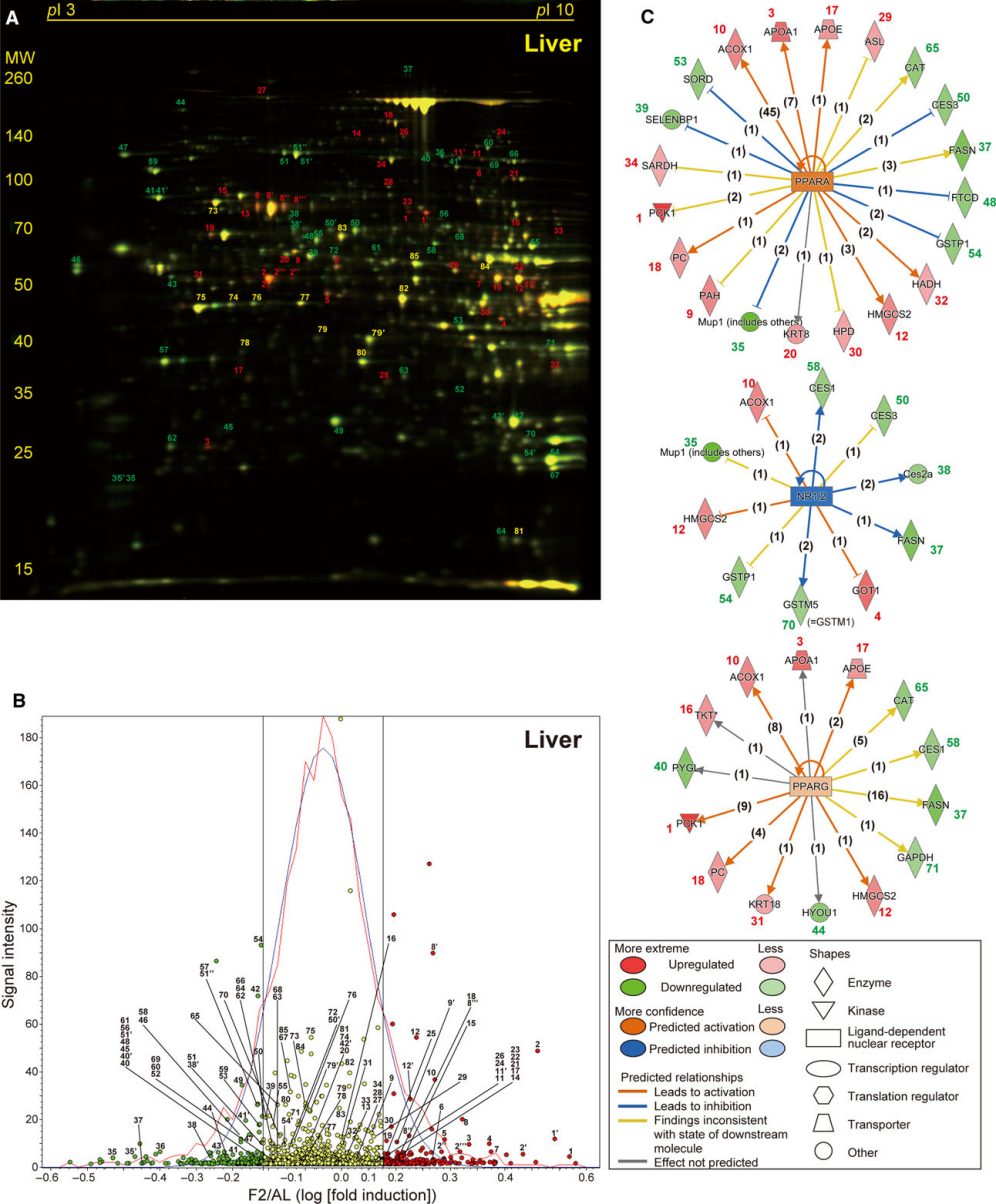
Fasting‐induced protein remodeling in the liver. Fluorescent 2D DIGE was performed on liver homogenates from *ad libitum*‐fed (AL) and 2‐day fasted (F2) mice. (A) Representative fluorescent gel image in which proteins upregulated by fasting are labeled in red and those downregulated are in green. Approximate isoelectric points (p*I* ) and molecular weights (MW; kDa) are indicated. (B) Quantitative profiling of the above image using DeCyder software. Upregulated and downregulated (> 1.1‐fold) protein spots are labeled in red and green, respectively, with others in yellow. The *x*‐axis represents log [(Fasted/AL) fold induction], and the *y*‐axis represents spot signal intensity; the red line represents spot number distribution, while the blue line its Gaussian approximation; and two black straight lines represent 1.1 and –1.1 fold change. (C) The three highest scoring upstream regulators (PPARα, NR1I2, and PPARγ) listed by Ingenuity Pathway Analysis (IPA) of the samples from AL and F2 mice (*n* = 4 each). Upregulated proteins with IDs identical to those in A and B are shown in red and downregulated proteins are in green, and predicted relationships are indicated by various types of lines described in the panel. The numbers in parentheses are the numbers of current publications reporting those relationships.

We reported previously that 2‐day fasting of adult C57BL/6J male mice causes 23.4% body and 32.2% liver weight losses 20. Despite such drastic alterations, the marked activation of protein degradation systems such as ubiquitin‐proteasome and autophagy‐lysosome systems was not detectable by RT‐PCR analysis of the liver, which highly contrasted with thymus, another highly atrophied (54.7% weight loss) organ 20. However, our 2D DIGE proteomic analyses revealed substantial numbers of proteins were up‐ or downregulated in the liver of F2 mice; for example, among a total of 1824 protein spots identified in the representative 2D gel (Fig. 2A), 214 (11.7%, red circles) and 178 (9.8%, green circles) spots were > 1.1‐fold up‐ and downregulated, respectively (Fig. 2B). By comparative analysis between the four independent gels using DeCyder software, we identified 34 significantly upregulated proteins (*P* < 0.05, Table 1 and Table S1 [Sheet A] for weblinks); these include key enzymes in gluconeogenesis (phosphoenolpyruvate carboxykinase, cytosolic [Pck1 (spot 1), the rate‐limiting enzyme of gluconeogenesis] and pyruvate carboxylase, mitochondrial [Pc (spot 18)]), peroxisomal fatty acid β‐oxidation (peroxisomal acyl‐CoA oxidase 1 [Acox1 (spot 10)]), ketogenesis (hydroxymethylglutaryl‐CoA synthase, mitochondrial [Hmgcs2 (spot 12, 12′), the rate‐limiting enzyme of ketogenesis] 21, 22), phenylalanine metabolism (phenylalanine‐4‐hydroxylase [Pah (spot 9)] and homogentisate 1,2‐dioxygenase [Hgd (spot 7)]), and *S*‐adenosylmethionine (the major methyl donor for hundreds of methyltransferases) synthesis (*S*‐adenosylmethionine synthase isoform type‐1 [Mat1a (spots 2, 2′, 2′′, 2′′′)]). We also identified 38 significantly downregulated proteins involved in fatty acid synthesis (fatty acid synthase [Fasn (spot 37)]), glycogenolysis (glycogen phosphorylase, liver form [Pygl (spot 40)], and glutathione conjugation (glutathione *S*‐transferases, π1 [Gstp1 (spot 54)]), and μ1 [Gstm1 (spot 70)] (Table 1 and Table S1 [Sheet A]).

**Table 1:** Protein expression changes upon 48‐h fasting in mouse liver

Liver Spot ID	Fold change	*P*‐value	Protein name	Uniprot ID	Gene name	Unigene ID	Mascot score	Sequence coverage	Peptide matches	MW	p*I*
Upregulated proteins											
1	2.89	0.003	Phosphoenolpyruvate carboxykinase, cytosolic (GTP)	Q9Z2V4	Pck1	Mm.266867	132	50%	30/90	70 051	6.18
1′	2.83	0.003	Phosphoenolpyruvate carboxykinase, cytosolic (GTP)	Q9Z2V4	Pck1	Mm.266867	181	50%	27/55	70 051	6.18
2	2.66	0.003	S‐adenosylmethionine synthase isoform type‐1	Q91X83	Mat1a	Mm.14064	76	24%	9/32	44 051	5.51
2′	2.51	0.003	S‐adenosylmethionine synthase isoform type‐1	Q91X83	Mat1a	Mm.14064	64	35%	13/83	44 051	5.51
3	2.03	0.003	Apolipoprotein A‐I	Q00623	Apoa1	Mm.26743	172	48%	16/23	30 597	5.51
4	2.01	0.011	Aspartate aminotransferase, cytoplasmic	P05201	Got1	Mm.19039	211	60%	24/46	46 504	6.68
5	1.97	0.010	Ornithine aminotransferase, mitochondrial	P29758	Oat	Mm.13694	147	53%	24/95	48 723	6.19
2′′	1.94	0.009	S‐adenosylmethionine synthase isoform type‐1	Q91X83	Mat1a	Mm.14064	106	28%	10/17	44 051	5.51
6	1.93	0.003	Dimethylglycine dehydrogenase, mitochondrial	Q9DBT9	Dmgdh	Mm.21789	109	27%	15/32	97 422	7.69
7	1.92	0.005	Homogentisate 1,2‐dioxygenase	Q05BJ1	Hgd	Mm.157442	61	17%	8/31	50 726	6.86
8	1.89	0.011	Serum albumin	P07724	Alb	Mm.16773	168	29%	15/17	70 700	5.75
8′	1.87	0.025	Serum albumin	P07724	Alb	Mm.16773	157	52%	33/113	70 700	5.75
2′′′	1.85	0.004	S‐adenosylmethionine synthase isoform type‐1	Q91X83	Mat1a	Mm.14064	65	22%	7/15	44 051	5.51
8′′	1.80	0.023	Serum albumin	P07724	Alb	Mm.16773	61	18%	9/25	70 700	5.75
9	1.63	0.010	Phenylalanine‐4‐hydroxylase	P16331	Pah	Mm.342177	78	28%	12/36	52 381	5.91
10	1.62	0.003	Peroxisomal acyl‐coenzyme A oxidase 1	Q9R0H0	Acox1	Mm.356689	73	27%	13/39	75 000	8.64
11	1.59	0.022	Elongation factor 2	P58252	Eef2	Mm.326799	153	47%	33/74	96 222	6.41
12	1.58	0.003	Hydroxymethylglutaryl‐CoA synthase, mitochondrial	P54869	Hmgcs2	Mm.289131	103	25%	13/29	57 300	8.65
13	1.55	0.013	Annexin A6	P14824	Anxa6	Mm.265347	85	26%	15/32	76 294	5.34
14	1.54	0.004	Alpha‐aminoadipic semialdehyde synthase, mitochondrial	Q99K67	Aass	Mm.18651	71	36%	23/86	103 650	6.42
15	1.51	0.003	Stress‐70 protein, mitochondrial	P38647	Hspa9	Mm.209419	104	35%	17/43	73 701	5.81
8′′′	1.50	0.034	Serum albumin	P07724	Alb	Mm.16773	171	54%	24/78	70 700	5.75
12′	1.44	0.004	Hydroxymethylglutaryl‐CoA synthase, mitochondrial	P54869	Hmgcs2	Mm.289131	101	39%	16/46	57 300	8.65
16	1.44	0.004	Transketolase	Q62371	Tkt	Mm.290692	72	20%	9/28	68 272	7.23
17	1.40	0.008	Apolipoprotein E	P08226	Apoe	Mm.305152	98	38%	15/32	35 901	5.56
9′	1.35	0.003	Phenylalanine‐4‐hydroxylase	P16331	Pah	Mm.342177	88	28%	11/35	52 381	5.91
18	1.35	0.004	Pyruvate carboxylase, mitochondrial	P06802	Pc	Mm.342177	120	16%	15/20	130 344	6.25
19	1.35	0.008	60 kDa heat shock protein, mitochondrial	P63038	Hspd1	Mm.1777	72	33%	15/48	61 088	5.91
20	1.35	0.016	Keratin, type II cytoskeletal	P11679	Krt8	Mm.358618	146	35%	18/26	54 531	5.70
21	1.31	0.016	Aconitate hydratase, mitochondrial	Q99KI0	Aco2	Mm.154581	93	13%	9/15	86 151	8.08
22	1.28	0.022	4‐aminobutyrate aminotransferase, mitochondrial	P61922	Abat	Mm.259315	58	25%	11/40	57 100	8.35
23	1.27	0.004	Succinate dehydrogenase (ubiquinone) flavoprotein subunit, mitochondrial	Q8K2B3	Sdha	Mm.158231	216	61%	28/59	73 623	7.06
24	1.27	0.013	Dihydropyrimidine dehydrogenase [NADP(+)]	Q80XT4	Dpyd	Mm.27907	90	20%	16/33	113 177	7.13
25	1.27	0.018	3‐mercaptopyruvate sulfurtransferase	Q99J99	Mpst	Mm.294215	64	32%	9/27	33 231	6.11
26	1.25	0.003	2‐oxoglutarate dehydrogenase, mitochondrial	Q60597	Ogdh	Mm.276348	80	17%	18/44	117 572	6.36
27	1.25	0.003	Clathrin heavy chain 1	Q68FD5	Cltc	Mm.254588	58	6%	12/18	193 202	5.48
28	1.24	0.013	Keratin, type I cytoskeletal 12	Q64291	Krt12	Mm.436651	63	21%	7/106	52 774	4.76
29	1.23	0.016	Argininosuccinate lyase	Q91YI0	Asl	Mm.23869	192	56%	29/71	51 878	6.48
30	1.23	0.039	4‐hydroxyphenylpyruvate dioxygenase	P49429	Hpd	Mm.439709	239	72%	32/95	45 254	6.58
11′	1.21	0.022	Elongation factor 2	P58252	Eef2	Mm.326799	152	43%	28/55	96 222	6.41
31	1.19	0.036	Keratin, type I cytoskeletal	P05784	Krt18	Mm.22479	133	35%	16/24	47 509	5.22
32	1.16	0.003	Hydroxyacyl‐coenzyme A dehydrogenase, mitochondrial	O08756	Hadh	Mm.260164	61	24%	7/29	34 613	8.76
33	1.16	0.005	Apoptosis‐inducing factor 1, mitochondrial	Q9Z0X1	Aifm1	Mm.240434	63	16%	8/19	66 952	9.23
34	1.14	0.020	Sarcosine dehydrogenase, mitochondrial	Q99LB7	Sardh	Mm.278467	58	20%	18/57	102 644	6.28
Downregulated proteins											
35	−2.58	0.044	Major urinary protein 2	P11589	Mup2	Mm.457980	152	77%	17/36	20 935	5.04
36	−2.38	0.003	DENN domain‐containing protein 2A	B7ZP28	Dennd2a	Mm.440021	57	8%	9/19	113 936	9.07
35′	−2.01	0.027	Major urinary protein 2	P11589	Mup2	Mm.457980	79	43%	8/23	20 935	5.04
37	−1.88	0.008	Fatty acid synthase	P19096	Fasn	Mm.236443	227	36%	85/131	274 994	6.13
38	−1.66	0.030	Pyrethroid hydrolase Ces2a	Q8QZR3	Ces2a	Mm.212983	188	58%	27/59	62 356	5.74
39	−1.64	0.014	Selenium‐binding protein 2	Q63836	Selenbp2	Mm.225405	68	18%	10/31	53 147	5.78
40	−1.63	0.003	Glycogen phosphorylase, liver form	Q9ET01	Pygl	Mm.256926	66	14%	11/21	97 857	6.63
41	−1.60	0.040	78 kDa glucose‐regulated protein	P20029	Hspa5	Mm.330160	202	41%	24/36	72 492	5.07
42	−1.60	0.010	Carbonic anhydrase 3	P16015	Car3	Mm.300	86	41%	8/19	29 633	6.89
43	−1.60	0.007	26S protease regulatory subunit 6B	P54775	Psmc4	Mm.29582	130	68%	21/95	47 493	5.09
44	−1.53	0.005	Hypoxia upregulated protein 1	Q9JKR6	Hyou1	Mm.116721	145	17%	19/22	111 340	5.12
45	−1.50	0.017	NADH dehydrogenase (ubiquinone) flavoprotein 2, mitochondrial	Q9D6J6	Ndufv2	Mm.2206	58	36%	6/23	27 610	7.00
46	−1.47	0.016	Calreticulin	P14211	Calr	Mm.1971	87	22%	9/17	48 136	4.33
41′	−1.46	0.019	78 kDa glucose‐regulated protein	P20029	Hspa5	Mm.330160	64	16%	10/23	72 492	5.07
47	−1.46	0.003	Endoplasmin	P08113	Hsp90b1	Mm.87773	57	13%	12/21	92 703	4.74
48	−1.44	0.017	Formimidoyltransferase‐cyclodeaminase	Q91XD4	Ftcd	Mm.36278	78	41%	17/73	59 529	5.79
49	−1.44	0.006	Indolethylamine *N*‐methyltransferase	P40936	Inmt	Mm.299	108	48%	13/46	30 068	6.00
46′	−1.43	0.024	Calreticulin	P14211	Calr	Mm.1971	80	29%	12/36	48 136	4.33
50	−1.43	0.022	Carboxylesterase 3A	Q63880	Ces3a	Mm.295534	94	23%	14/39	63 677	5.78
40′	−1.42	0.017	Glycogen phosphorylase, liver form	Q9ET01	Pygl	Mm.256926	169	35%	24/36	97 857	6.63
51	−1.42	0.016	Cytosolic 10‐formyltetrahydrofolate dehydrogenase	Q8R0Y6	Aldh1 l1	Mm.30035	62	9%	7/9	99 502	5.64
51′	−1.41	0.003	Cytosolic 10‐formyltetrahydrofolate dehydrogenase	Q8R0Y6	Aldh1 l1	Mm.30035	338	76%	54/97	99 502	5.64
52	−1.39	0.016	Putative l‐aspartate dehydrogenase	Q9DCQ2	Aspdh	Mm.88478	66	39%	9/38	30 479	6.45
53	−1.39	0.009	Sorbitol dehydrogenase	Q64442	Sord	Mm.371580	108	35%	9/22	38 795	6.56
54	−1.38	0.016	Glutathione *S*‐transferase π1	P19157	Gstp1	Mm.299292	73	44%	10/42	23 765	7.68
51′′	−1.37	0.004	Cytosolic 10‐formyltetrahydrofolate dehydrogenase	Q8R0Y6	Aldh1 l1	Mm.30035	235	38%	28/37	99 502	5.64
55	−1.36	0.006	Protein disulfide‐isomerase A3	P27773	Pdia3	Mm.263177	141	41%	19/43	57 099	5.88
54′	−1.33	0.009	Glutathione *S*‐transferase π1	P19157	Gstp1	Mm.299292	87	53%	11/47	23 765	7.68
56	−1.32	0.005	DDRGK domain‐containing protein 1	Q80WW9	Ddrgk1	Mm.440063	55	21%	5/15	35 956	5.32
57	−1.32	0.037	Regucalcin	Q64374	Rgn	Mm.2118	192	71%	20/55	33 899	5.15
58	−1.32	0.014	Carboxylesterase 1D	Q8VCT4	Ces1d	Mm.292803	110	36%	17/67	62 034	6.17
59	−1.31	0.009	Heat shock protein HSP 90‐beta	P11499	Hsp90ab1	Mm.2180	89	19%	13/27	83 571	4.97
38′	−1.30	0.023	Pyrethroid hydrolase Ces2a	Q8QZR3	Ces2a	Mm.212983	63	13%	6/8	62 356	5.74
60	−1.29	0.034	C‐1‐tetrahydrofolate synthase, cytoplasmic	Q922D8	Mthfd1	Mm.29584	168	43%	32/71	101 820	6.70
61	−1.29	0.005	T‐complex protein 1 subunit beta	P80314	Cct2	Mm.247788	58	20%	9/26	57 783	5.97
62	−1.28	0.010	Lactoylglutathione lyase	Q9CPU0	Glo1	Mm.261984	99	66%	13/87	20 967	5.24
63	−1.26	0.005	3‐hydroxyanthranilate 3,4‐dioxygenase	Q78JT3	Haao	Mm.30100	105	33%	10/18	32 955	6.09
50′	−1.24	0.026	Carboxylesterase 3A	Q63880	Ces3a	Mm.295534	61	16%	8/21	63 677	5.78
42′	−1.24	0.019	Carbonic anhydrase 3	P16015	Car3	Mm.300	85	63%	15/83	29 633	6.89
64	−1.23	0.003	Nucleoside diphosphate kinase B	Q01768	Nme2	Mm.1260	62	59%	7/32	17 466	6.97
65	−1.22	0.023	Catalase	P70423	Cat	Mm.4215	115	22%	12/21	60 043	7.72
66	−1.21	0.039	Cytoplasmic aconitate hydratase	P28271	Aco1	Mm.331547	223	52%	37/77	98 691	7.23
67	−1.20	0.009	Peroxiredoxin‐1	P35700	Prdx1	Mm.30929	90	47%	9/27	22 390	8.26
68	−1.18	0.038	Acyl‐CoA synthetase family member 2, mitochondrial	Q8VCW8	Acsf2	Mm.386885	134	49%	25/96	68 591	8.44
69	−1.18	0.011	Methylmalonyl‐CoA mutase, mitochondrial	P16332	Mut	Mm.259884	79	36%	19/64	83 248	6.45
70	−1.18	0.009	Glutathione *S*‐transferase μ1	P10649	Gstm1	Mm.37199	169	77%	21/49	26 067	7.71
71	−1.14	0.027	Glyceraldehyde‐3‐phosphate dehydrogenase	P16858	Gapdh	Mm.304088	75	50%	16/68	36 072	8.44
72	−1.11	0.034	Selenium‐binding protein 1	P17563	Selenbp1	Mm.196558	104	25%	12/21	53 051	5.87

Proteomic data analysis using IPA software revealed PPARα (= PPARA) as the highest scoring upstream regulator, followed by nuclear receptor subfamily 1 group I member 2 (NR1I2; also known as PXR [pregnane X receptor]) and peroxisome proliferator‐activated receptor gamma (PPARG = PPARγ) (Table S2 [Sheet A]). Among multiple PPARα‐regulated proteins 23, 24, a total of 20 up‐ or downregulated proteins were identified (Fig. 2C). Moreover, among NR1I2‐ and PPARγ‐regulated proteins, 10 and 14 up‐ or downregulated proteins were identified, respectively (Fig. 2C), some of which might contribute to their relatively high scores (Table S2 [Sheet A]). In addition, TP53 was listed as the fourth highest scoring regulator in the liver by regulating expression of 19 proteins (Table S2 [Sheet A]).

### Protein expression changes in the kidney

**Figure 3:**
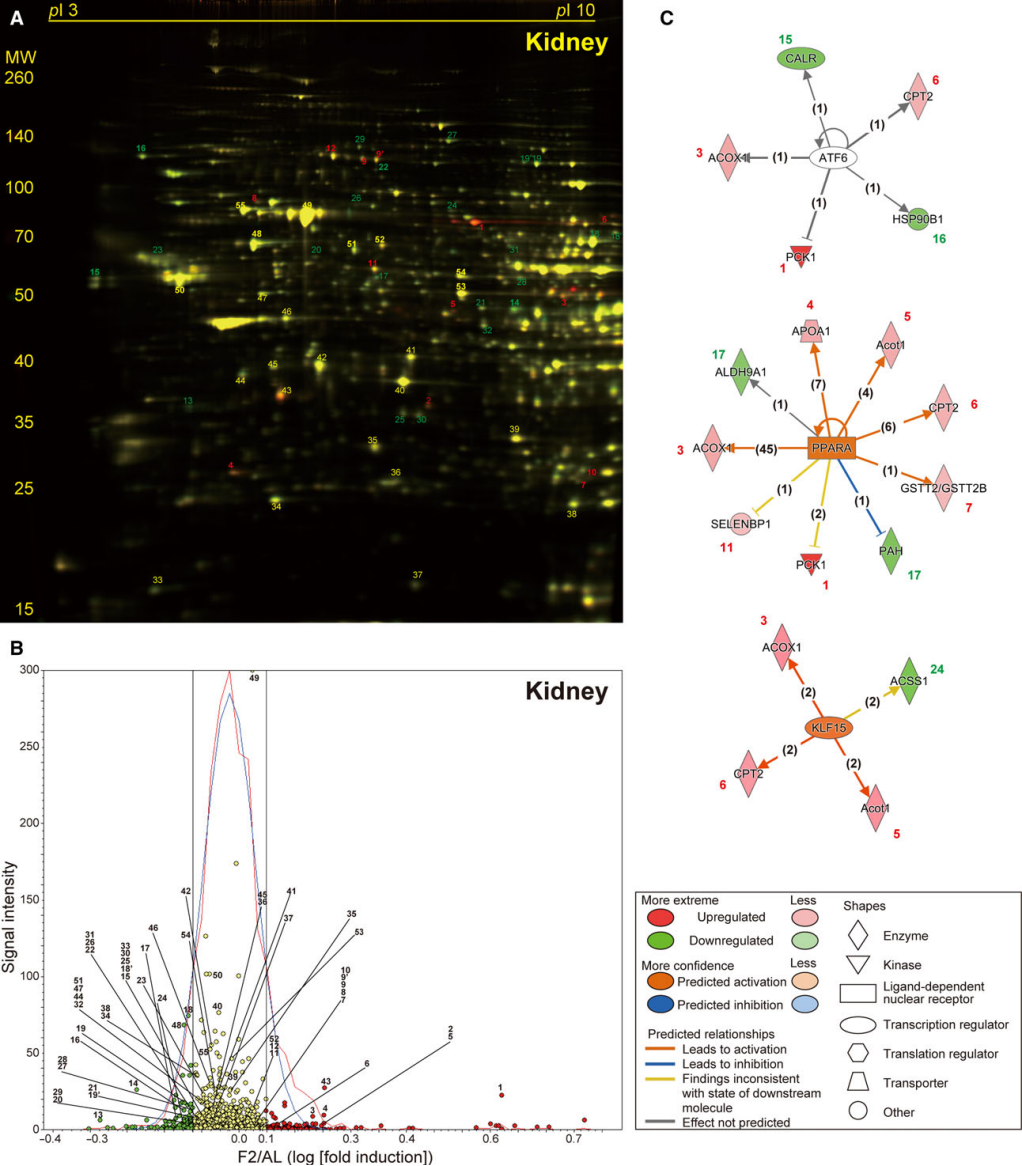
Fasting‐induced protein remodeling in the kidney. Fluorescent 2D DIGE was performed on kidney homogenates from *ad libitum*‐fed (AL) and 2‐day fasted (F2) mice. (A) Representative fluorescent gel image in which proteins upregulated by fasting are labeled in red and those downregulated are in green. (B) Quantitative profiling of the above image using DeCyder. (C) The three highest scoring upstream regulators (ATF6, PPARα, and KLF15) listed by IPA of the samples from AL and F2 mice (*n* = 4 each). Refer to Fig. 2 legend for detailed information.

Two‐day fasting induced 18.6% kidney weight loss with no or mild activation of protein degradation systems in our previous RT‐PCR analysis 20. Proteomic analysis revealed a total of 1633 spots in the representative 2D gel (Fig. 3A), of which 45 (2.76%, red circles) and 44 (2.69%, green circles) spots were > 1.1‐fold up‐ and downregulated, respectively (Fig. 3B). We identified only 12 significantly upregulated proteins in the kidney of F2 mice, which included Pck1 (spot 1), Acox1 (spot 3), and apolipoprotein A‐I (Apoa1, spot 4), just like in the liver (Table 2 and Table S1 [Sheet A and B]). We also identified 20 significantly downregulated proteins in the kidney; most of them (except calreticulin [Calr (spot 15)] and endoplasmin [Hsp90b1 (heat shock protein 90 kDa beta member 1, spot 16)]) were not apparently changed in the liver (Table 2 and Table S1 [Sheet A and B]). Nevertheless, the upstream regulator analysis revealed PPARα as the second highest scoring upstream regulator after ATF6 (activating transcriptional factor 6) and followed by KLF15 (Krüppel‐like factor 15) (Fig. 3C and Table S2 [Sheet B]).

**Table 2:** Protein expression changes upon 48‐h fasting in mouse kidney

Kidney spot ID	Fold change	*P*‐VALUE	Protein name	Uniprot ID	Gene name	Unigene ID	Mascot score	Sequence coverage	Peptide matches	MW	p*I*
Upregulated proteins											
1	3.67	0.004	Phosphoenolpyruvate carboxykinase, cytosolic (GTP)	Q9Z2V4	Pck1	Mm.266867	183	53%	31/77	70 051	6.18
2	1.59	0.004	*N*‐acetylglucosaminyl‐phosphatidylinositol de‐*N*‐acetylase	Q5SX19	Pigl	Mm.390358	61	39%	7/28	28 450	8.59
3	1.50	0.014	Peroxisomal acyl‐coenzyme A oxidase 1	Q9R0H0	Acox1	Mm.356689	121	33%	15/29	75 000	8.64
4	1.48	0.018	Apolipoprotein A‐I	Q00623	Apoa1	Mm.26743	111	39%	14/35	30 597	5.51
5	1.43	0.011	Mixture: Acyl‐coenzyme A thioesterase 1	O55137	Acot1	Mm.1978	79	39%	11/38	46 335	6.12
			Mixture: Acyl‐coenzyme A thioesterase 2	Q9QYR9	Acot2	Mm.371675	65	32%	10/38	49 854	6.88
6	1.32	0.028	Carnitine O‐palmitoyltransferase 2, mitochondrial	P52825	Cpt2	Mm.307620	59	27%	12/44	74 504	8.59
7	1.19	0.018	Glutathione *S*‐transferase τ2	Q61133	Gstt2	Mm.24118	87	41%	9/26	27 731	7.02
8	1.14	0.034	Stress‐70 protein, mitochondrial	P38647	Hspa9	Mm.209419	106	46%	24/73	73 701	5.81
9	1.14	0.004	Villin‐1	Q62468	Vil1	Mm.471601	155	27%	20/28	93 230	5.72
9′	1.14	0.017	Villin‐1	Q62468	Vil1	Mm.471601	159	35%	27/44	93 230	5.72
10	1.12	0.027	Glutathione *S*‐transferase μ1	P10649	Gstm1	Mm.37199	120	69%	17/50	26 067	7.71
11	1.11	0.034	Selenium‐binding protein 1	P17563	Selenbp1	Mm.196558	208	51%	20/29	53 051	5.87
12	1.11	0.044	Cytosolic 10‐formyltetrahydrofolate dehydrogenase	Q8R0Y6	Aldh1 l1	Mm.30035	238	49%	37/65	99 502	5.64
Downregulated proteins											
13	−1.84	0.004	Inositol oxygenase	Q9QXN5	Miox	Mm.158200	108	55%	13/47	33 484	5.03
14	−1.56	0.004	Glycine amidinotransferase, mitochondrial	Q9D964	Gatm	Mm.29975	150	32%	16/30	48 779	8.00
15	−1.32	0.035	Calreticulin	P14211	Calr	Mm.1971	100	27%	13/30	48 136	4.33
16	−1.31	0.004	Endoplasmin	P08113	Hsp90b1	Mm.87773	74	14%	14/21	92 703	4.74
17	−1.29	0.023	Phenylalanine‐4‐hydroxylase	P16331	Pah	Mm.263539	69	31%	13/50	52 381	5.91
18	−1.28	0.027	Acyl‐coenzyme A synthetase ACSM2, mitochondrial	Q8K0L3	Acsm2	Mm.268448	215	40%	22/28	64 741	8.54
19	−1.26	0.014	Elongation factor 2	P58252	Eef2	Mm.326799	65	12%	9/13	96 222	6.41
20	−1.22	0.027	Sulfite oxidase, mitochondrial	Q8R086	Suox	Mm.23352	62	14%	7/15	61 231	6.07
21	−1.20	0.009	Elongation factor Tu, mitochondrial	Q8BFR5	Tufm	Mm.197829	70	16%	8/14	49 876	7.23
22	−1.19	0.050	Gelsolin	P13020	Gsn	Mm.21109	72	26%	12/38	86 287	5.83
18′	−1.19	0.019	Acyl‐coenzyme A synthetase ACSM2, mitochondria	Q8K0L3	Acsm2	Mm.268448	114	49%	22/94	64 741	8.54
23	−1.18	0.049	Nucleolin	Q91VA3	Ncl	Mm.154378	61	10%	7/12	76 734	4.69
24	−1.18	0.023	Acetyl‐coenzyme A synthetase 2‐like, mitochondrial	Q99NB1	Acss1	Mm.7044	60	16%	10/27	75 317	6.51
25	−1.18	0.019	Beta‐lactamase‐like protein 2	Q99KR3	Lactb2	Mm.89572	81	34%	7/13	33 019	5.89
19′	−1.17	0.049	Elongation factor 2	P58252	Eef2	Mm.326799	60	17%	14/37	96 222	6.41
26	−1.17	0.043	Heat shock protein 75 kDa, mitochondrial	Q9CQN1	Trap1	Mm.123366	147	44%	25/49	80 501	6.25
27	−1.17	0.009	2‐oxoglutarate dehydrogenase, mitochondrial	Q60597	Ogdh	Mm.276348	155	24%	23/40	117 572	6.36
28	−1.16	0.049	4‐trimethylaminobutyraldehyde dehydrogenase	Q9JLJ2	Aldh9a1	Mm.330055	67	23%	10/27	54 449	6.63
29	−1.15	0.031	Lon protease homolog, mitochondrial	Q56A16	Lonp1	Mm.329136	63	15%	14/29	106 347	6.15
30	−1.13	0.039	3‐hydroxyisobutyrate dehydrogenase, mitochondrial	Q99L13	Hibadh	Mm.286458	78	21%	7/12	35 816	8.37
31	−1.13	0.018	EH domain‐containing protein 1	Q9WVK4	Ehd1	Mm.30169	60	21%	10/26	60 622	6.35
32	−1.12	0.034	Isovaleryl‐CoA dehydrogenase, mitochondrial	Q9JHI5	Ivd	Mm.6635	106	38%	13/27	46 695	8.53

### Protein expression changes in the thymus and spleen

**Figure 4:**
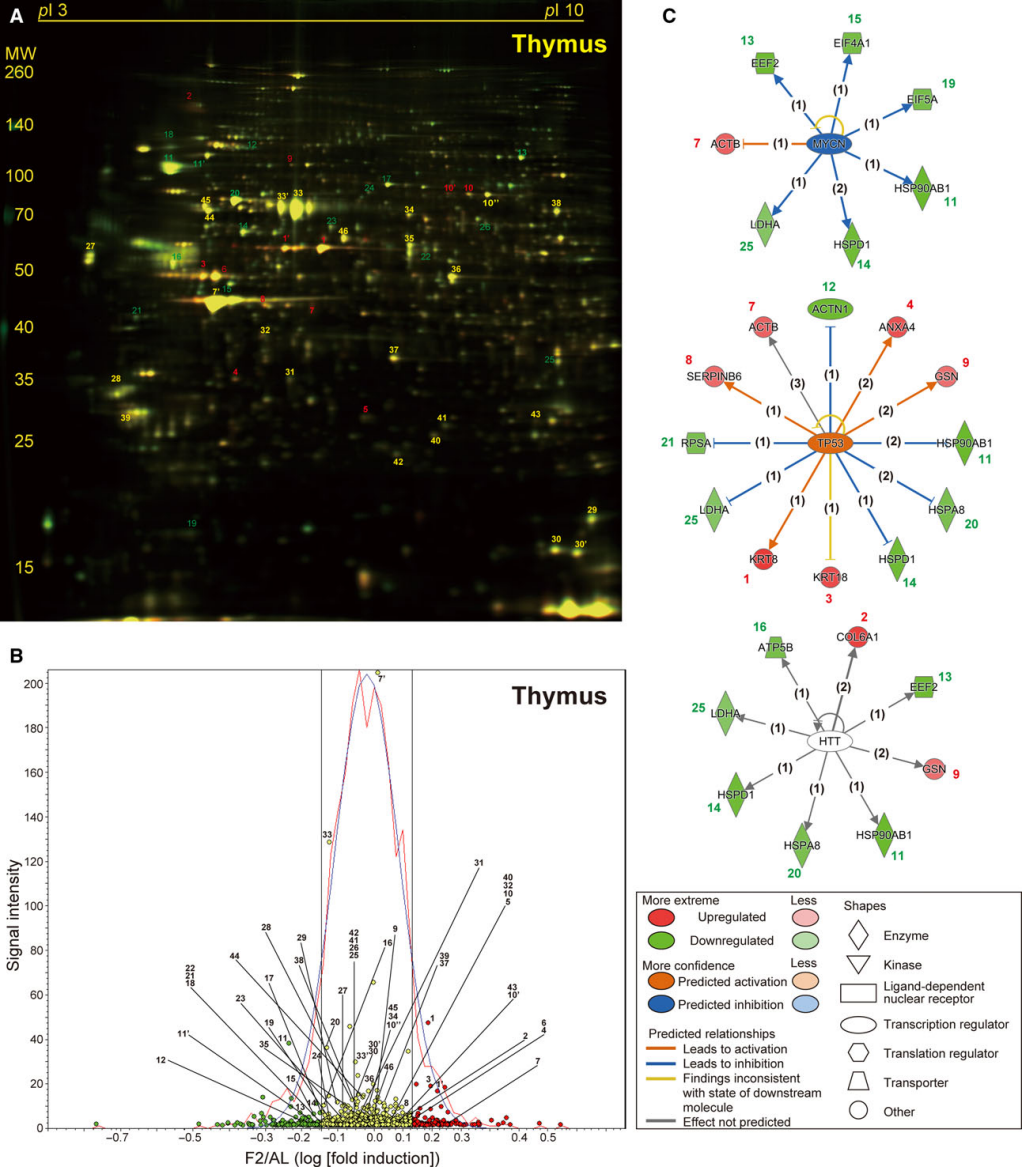
Fasting‐induced protein remodeling in the thymus. Fluorescent 2D DIGE was performed on thymus homogenates from *ad libitum*‐fed (AL) and 2‐day fasted (F2) mice. (A) Representative fluorescent gel image in which proteins upregulated by fasting are labeled in red and those downregulated are in green. (B) Quantitative profiling of the above image using DeCyder. (C) The three highest scoring upstream regulators (MYCN, TP53, and HTT) listed by IPA of the samples from AL and F2 mice (*n* = 4 each). Refer to Fig. 2 legend for detailed information.

**Figure 5:**
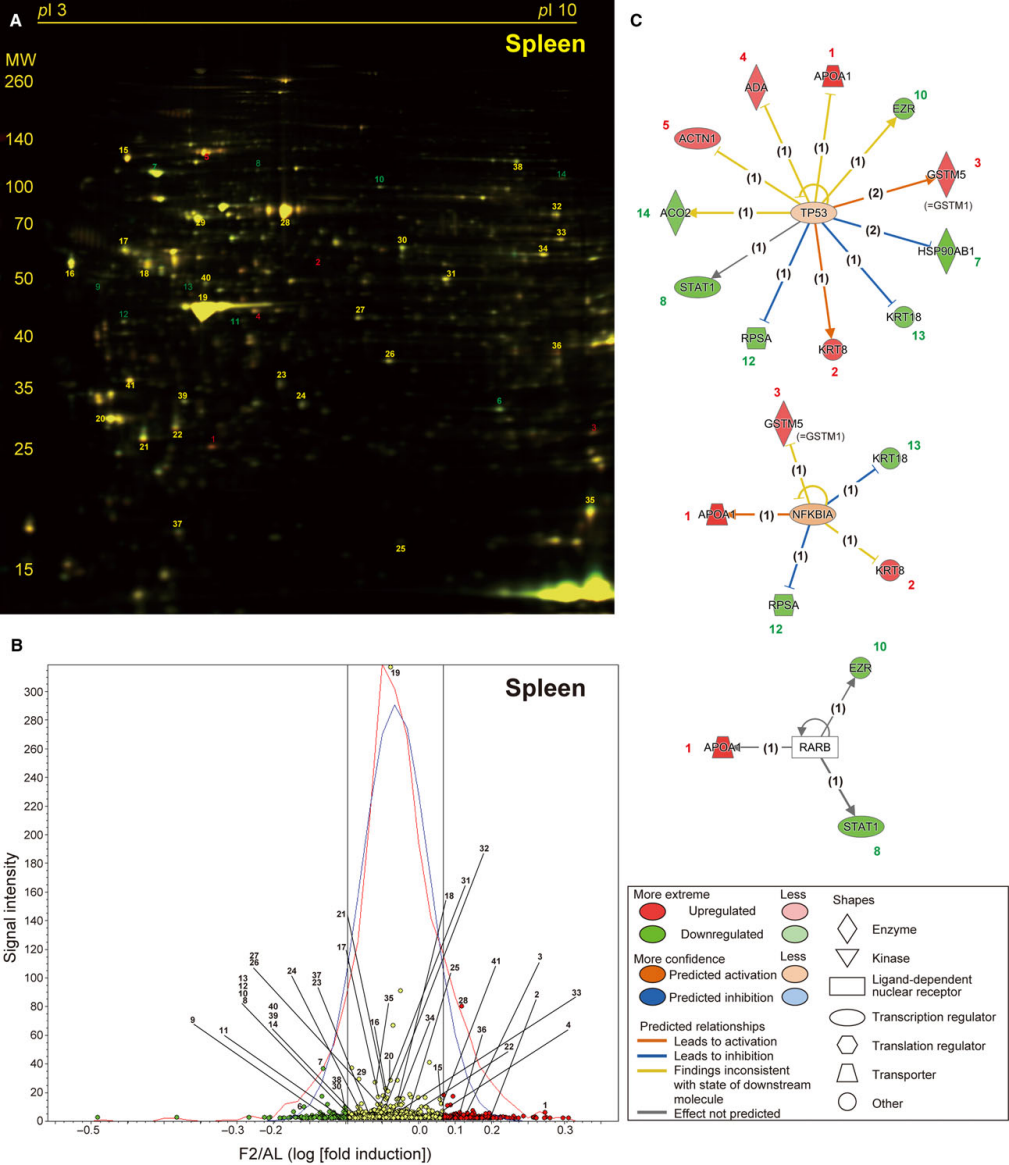
Fasting‐induced protein remodeling in the spleen. Fluorescent 2D DIGE was performed on spleen homogenates from *ad libitum*‐fed (AL) and 2‐day fasted (F2) mice. (A) Representative fluorescent gel image in which proteins upregulated by fasting are labeled in red and those downregulated are in green. (B) Quantitative profiling of the above image by DeCyder. (C) The three highest scoring upstream regulators (TP53, NFKBIA, and RARB) listed by IPA of the samples from AL and F2 mice (*n* = 4 each). Refer to Fig. 2 legend for detailed information.

Two‐day fasting induced marked weight loss in both the thymus and spleen (54.7% and 41.2%, respectively) although protein degradation systems were only found to be highly activated in the thymus but not in the spleen 20. In a representative 2D gel, among a total of 1874 spots detected in the thymus (Fig. 4A), 54 (2.88%, red circles) and 67 (3.57%, green circles) spots were found to be up‐ or downregulated, respectively (Fig. 4B). We identified 10 and 16 significantly up‐ and downregulated proteins in the thymus, respectively; the former includes several structural proteins such as keratin, collagen, annexin, actin, and moesin (Table 3 and Table S1 [Sheet C]). Upstream regulators included MYCN (*v*‐myc myelocytomatosis viral‐related oncogene, neuroblastoma derived [avian]), TP53, and huntingtin (HTT) in this order (Fig. 4C and Table S2 [Sheet C]). In a representative 2D gel, among a total of 1861 spots detected in the spleen (Fig. 5A), 42 (2.26%, red circles) and 34 (1.83%, green circles) spots were found to be up‐ or downregulated, respectively (Fig. 5B). We could identify only five and nine significantly up‐ and downregulated proteins in the spleen, respectively (Table 4 and Table S1 [Sheet D]), although the upstream regulators included TP53 with the highest score, followed by nuclear factor of NFKBIA (kappa light polypeptide gene enhancer in B‐cell inhibitor, alpha) and RARB (retinoic acid receptor, beta) (Fig. 5C and Table S2 [Sheet D]).

**Table 3:** Protein expression changes upon 48‐h fasting in mouse thymus

Thymus spot ID	Fold change	*P*‐value	Protein name ID	Uniprot name	Gene ID	Unigene score	Mascot	Sequence coverage	Peptide matches	MW	p*I*
Upregulated proteins											
1	1.82	0.031	Keratin, type II cytoskeletal 8	P11679	Krt8	Mm.358618	146	52%	30/66	54 531	5.70
2	1.71	0.031	Collagen alpha‐1 (VI) chain	Q04857	Col6a1	Mm.2509	109	40%	31/98	109 562	5.20
1′	1.70	0.042	Keratin, type II cytoskeletal 8	P11679	Krt8	Mm.358618	163	48%	24/43	54 531	5.70
3	1.61	0.018	Keratin, type I cytoskeletal 18	P05784	Krt18	Mm.22479	120	40%	17/41	47 509	5.22
4	1.54	0.024	Annexin A4	P97429	Anxa4	Mm.259702	85	61%	18/76	36 178	5.43
5	1.51	0.049	Proteasome subunit beta type‐10	O35955	Psmb10	Mm.787	72	56%	9/54	29 330	6.40
6	1.38	0.020	Heterogeneous nuclear ribonucleoprotein F	Q9Z2X1	Hnrnpf	Mm.422979	122	53%	15/64	46 043	5.31
7	1.37	0.049	Actin, cytoplasmic 1	P60710	Actb	Mm.391967	63	57%	12/65	42 052	5.29
8	1.29	0.018	Serpin B6	Q60854	Serpinb6	Mm.252210	59	40%	13/67	42 913	5.53
9	1.24	0.050	Gelsolin	P13020	Gsn	Mm.21109	85	34%	19/71	86 287	5.83
10	1.21	0.050	Moesin	P26041	Msn	Mm.138876	124	48%	29/71	67 839	6.22
10′	1.17	0.049	Moesin	P26041	Msn	Mm.138876	59	45%	25/122	67 839	6.22
Downregulated proteins											
11	−1.68	0.018	Heat shock protein HSP 90‐beta	P11499	Hsp90ab1	Mm.2180	152	32%	19/24	83 571	4.97
12	−1.62	0.018	Alpha‐actinin‐1	Q7TPR4	Actn1	Mm.253564	116	47%	38/119	103 631	5.23
13	−1.59	0.028	Elongation factor 2	P58252	Eef2	Mm.326799	78	17%	14/26	96 222	6.41
14	−1.54	0.035	60 kDa heat shock protein, mitochondrial	P63038	Hspd1	Mm.1777	70	18%	9/16	61 088	5.91
11′	−1.49	0.050	Heat shock protein HSP 90‐beta	P11499	Hsp90ab1	Mm.2180	121	50%	30/103	83 571	4.97
15	−1.40	0.018	Eukaryotic initiation factor 4A‐I	P60843	Eif4a1	Mm.371557	86	30%	12/23	46 353	5.32
16	−1.36	0.050	ATP synthase subunit beta, mitochondrial	P56480	Atp5b	Mm.238973	203	50%	22/37	56 265	5.19
13′	−1.35	0.024	Elongation factor 2	P58252	Eef2	Mm.326799	173	45%	32/72	96 222	6.41
17	−1.35	0.039	Mitochondrial import receptor subunit TOM34	Q9CYG7	Tomm34	Mm.23173	55	39%	10/86	34 656	9.24
18	−1.34	0.020	Heterogeneous nuclear ribonucleoprotein U‐like protein 2	Q00PI9	Hnrnpul2	Mm.476519	69	34%	23/91	85 515	4.83
19	−1.33	0.047	Eukaryotic translation initiation factor 5A‐1	P63242	Eif5a	Mm.29324	84	60%	9/67	17 049	5.08
20	−1.30	0.031	Heat shock cognate 71 kDa protein	P63017	Hspa8	Mm.290774	76	20%	13/32	71 055	5.37
21	−1.30	0.049	40S ribosomal protein SA	P14206	Rpsa	Mm.4071	105	46%	13/68	32 931	4.80
22	−1.29	0.049	Fibrinogen beta chain	Q8K0E8	Fgb	Mm.30063	76	42%	16/66	55 402	6.68
23	−1.25	0.035	T‐complex protein 1 subunit epsilon	P80316	Cct5	Mm.282158	98	31%	14/26	60 042	5.72
24	−1.22	0.018	Glycine‐tRNA ligase	Q9CZD3	Gars	Mm.250004	97	40%	24/96	82 624	6.24
25	−1.11	0.043	l‐lactate dehydrogenase A chain	P06151	Ldha	Mm.29324	98	30%	10/19	36 817	7.62
26	−1.10	0.042	Bifunctional purine biosynthesis protein PURH	Q9CWJ9	Atic	Mm.38010	132	54%	28/91	64 690	6.30

**Table 4:** Protein expression changes upon 48‐h fasting in mouse spleen

Spleen spot ID	Fold change	*P*‐ value	Protein name	Uniprot ID	Gene name	Unigene ID	Mascot score	Sequence coverage	Peptide matches	MW	p*I*
Upregulated proteins											
1	1.68	0.007	Apolipoprotein A‐I	Q00623	Apoa1	Mm.26743	120	51%	18/60	30 597	5.51
2	1.37	0.018	Keratin, type II cytoskeletal 8	P11679	Krt8	Mm.358618	193	70%	43/128	54 531	5.70
3	1.33	0.043	Glutathione *S*‐transferase μ1	P10649	Gstm1	Mm.37199	107	77%	24/107	26 067	7.71
4	1.23	0.020	Adenosine deaminase	P03958	Ada	Mm.388	64	55%	14/77	40 251	5.48
5	1.23	0.030	Mixture: Alpha‐actinin‐1	Q7TPR4	Actn1	Mm.253564	185	63%	50/132	103 631	5.23
			Mixture: Alpha‐actinin‐4	P57780	Actn4	Mm.81144	168	55%	44/132	105 368	5.25
Downregulated proteins											
6	−1.62	0.030	Carbonic anhydrase 2	P00920	Ca2	Mm.239871	70	60%	9/51	29 129	6.49
7	−1.36	0.007	Mixture: Heat shock protein HSP 90‐beta	P11499	Hsp90ab	Mm.222	191	58%	39/99	83 571	4.97
			Mixture: Heat shock protein HSP 90‐alpha	P07901	Hsp90aa1	Mm.341186	127	53%	32/99	85 134	4.93
8	−1.30	0.034	Signal transducer and activator of transcription 1	P42225	Stat1	Mm.277406	73	30%	18/59	87 826	5.42
9	−1.27	0.006	Ribonuclease inhibitor	Q91VI7	Rnh1	Mm.10152	133	68%	21/76	51 495	4.69
10	−1.23	0.030	Ezrin	P26040	Ezr	Mm.277812	118	57%	36/99	69 478	5.83
11	−1.21	0.030	Glutaredoxin‐3	Q9CQM9	Glrx3	Mm.267692	76	43%	12/75	38 039	5.42
12	−1.20	0.030	40S ribosomal protein SA	P14206	Rpsa	Mm.4071	147	58%	19/85	32 931	4.80
13	−1.16	0.012	Keratin, type I cytoskeletal 18	P05784	Krt18	Mm.22479	129	66%	24/85	47 509	5.22
14	−1.12	0.030	Aconitate hydratase, mitochondrial	Q99KI0	Aco2	Mm.154581	234	55%	40/106	86 151	8.08

**Figure 6:**
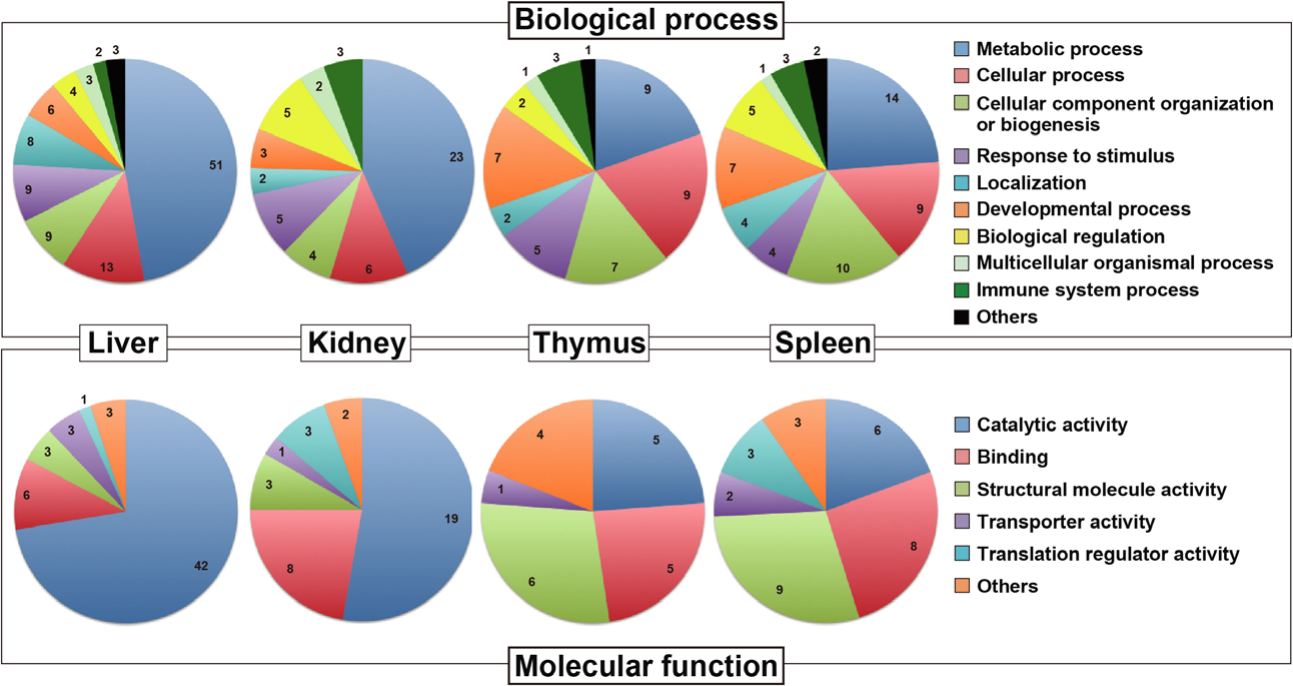
Venn diagrams for clarification of identified proteins that are involved in various biological processes and molecular functions. Fasting‐regulated proteins in the liver, kidney, thymus, and spleen are categorized by ‘biological process’ or ‘molecular function’ using PANTHER software.

When differentially expressed proteins (upon fasting) were classified by the biological processes involved and molecular functions using PANTHER software, both classification patterns were quite similar between the liver and kidney and between the thymus and spleen (Fig. 6).

### Protein expression changes in the brain and testis

**Figure 7:**
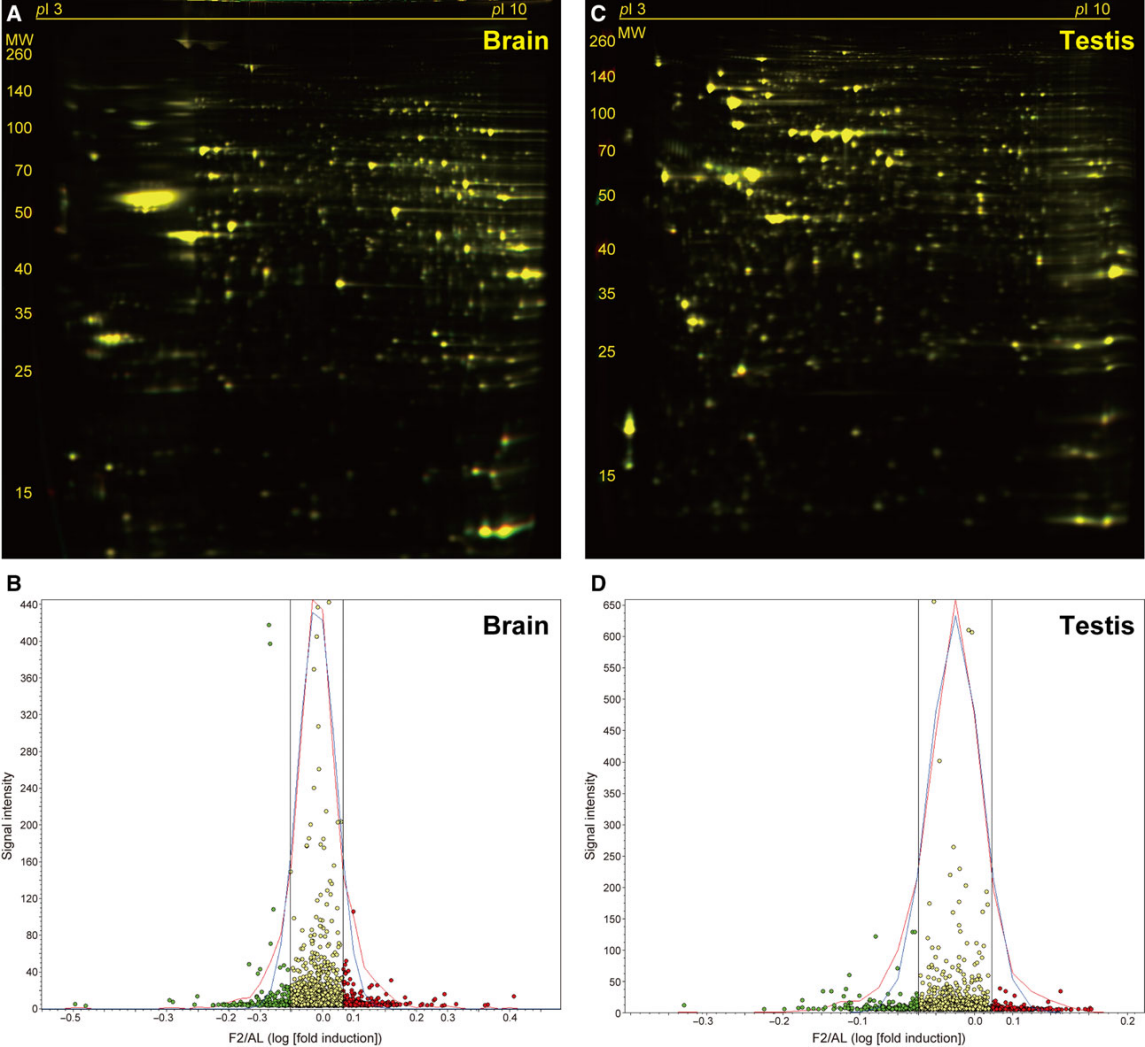
Fasting‐induced protein remodeling in the brain and testis. Fluorescent 2D DIGE was performed on brain (A and B) and testis (C and D) homogenates from *ad libitum*‐fed (AL) and 2‐day fasted (F2) mice. (A and C) Representative fluorescent images, in which proteins upregulated by fasting are labeled in red and those downregulated are in green. (B and D) Quantitative profiling of the above images using DeCyder software. Refer to Fig. 2 legend for detailed information.

Two‐day fasting induced only 2.7% and 9.2% weight losses in the brain and testis, respectively 20. No apparent protein degradation systems were found to be activated in either organ in our previous RT‐PCR analysis 20. In representative 2D gels, among a total of 2189 spots detected in the brain (Fig. 7A), 360 (16.4%, red circles) and 337 (15.4%, green circles) spots were found to be up‐ or downregulated, respectively (Fig. 7B), and among a total of 2301 spots detected in the testis (Fig. 7C), 184 (8.0%, red circles) and 249 (10.8%, green circles) spots were found to be up‐ or downregulated, respectively (Fig. 7D). However, the DeCyder comparative analysis of four AL and four F2 brain (and testis) samples revealed no consistent alterations in protein expression.

## Discussion

This study investigated global protein expression changes in six major mouse organs upon 2‐day fasting. After 1‐ or 2‐day fasting, blood levels of glucose, insulin, and C‐peptide 2 (another component of proinsulin [insulin + C‐peptide 2] that has its own physiological properties 25) were significantly reduced compared with the levels in AL mice (67.1%, 61.3%, and 66.3%, respectively [Fig. 1]). Moreover, the levels of two adipose‐derived peptide hormones, leptin (the pleiotropic hormone of satiation signals/energy expenditure 26, 27) and resistin (the hormone that may ‘resist’ insulin actions 28), were decreased to the same extent in F1 and F2 mice (Fig. 1). In contrast, ketone bodies and GIP became more accumulated in F2 mice (Fig. 1). High accumulation of ketone bodies may reflect a progressive systemic energy shift from glucose to ketone bodies. GIP is a polypeptide inhibitor of gastric acid secretion and acts as an affective promotor of insulin secretion 29; therefore, its elevation could be a delayed compensatory action against low plasma levels of insulin (Fig. 1). These data indicated somewhat altered systemic conditions between F1 and F2 mice. We identified 72, 26, 14, and 32 proteins that were significantly up‐ or downregulated in the liver, thymus, spleen, and kidney of F2 mice, respectively, but significant expression changes were not found in F1 mice (data not shown).

Previous studies demonstrated the pivotal roles of PPARα in mediating adaptive responses to fasting in the liver (Fig. 2C and Table S2 [Sheet A]), as observed in the present study; PPARα positively regulates gluconeogenesis, peroxisomal/mitochondrial β‐oxidation, fatty acid transport, and ketogenesis and also negatively regulates glycolysis, amino acid metabolism, and inflammation, by binding to specific nucleotide sequences known as peroxisome proliferator response elements (PPREs) in the promoter regions of target genes 23, 24. PPARα‐knockout mice have been shown to display several dysregulated responses such as severe hypoglycemia, hypoketonemia, elevated plasma free fatty acid levels, and fatty liver upon fasting 4, 5. Accordingly, we found hepatic upregulation of enzymes involved in gluconeogenesis (Pck1 and Pc), lipid β‐oxidation (Acox1), ketogenesis (Hmgcs2), and downregulation of the enzymes involved in fatty acid synthesis (Fasn) and glycogenolysis (Pygl) (Fig. 2C, Table 1, and Table S1 [Sheet A]). Although all genes encoding these ‘upregulated’ proteins have been shown to contain PPREs in their promoter regions 30, 31, 32, 33, 34 and thus can be activated directly by ligand‐bound PPARα, the suppression of Fasn and Pygl expression could be caused by a secondary mechanism such as low plasma levels of insulin 35, 36.

PPARα is activated by both endogenous and synthetic ligands; the former includes long‐chain polyunsaturated fatty acids and eicosanoids such as leukotriene B_4_ and the latter includes fibrates such as fenofibrate, bezafibrate, and clinofibrate, the drugs for the treatment of hypertriglyceridemia 24. Endogenous ligand activation of PPARα could occur in other PPARα‐expressing organs such as kidney and heart 37 because large amounts of free fatty acids enter the systemic circulation during fasting. Indeed, we found PPARα regulation of nine proteins in the kidney of F2 mice (Fig 3C and Table S2 [Sheet B]) although the regulatory genes and directions were not necessarily identical to the liver (Figs. 2C and 3C). The upstream regulator analysis listed NR1I2 and PPARγ as the second and third highest scoring transcriptional regulators, respectively, but their *P*‐values were much (7–8 orders) higher than PPARα (Fig. 2C and Table S2 [Sheet A]). Expression of PPARγ was rather restricted to adipose tissue and the immune systems, and its hepatic expression was shown to be extremely low compared with PPARα in adult rats 37.

The highest scoring regulator in the kidney of F2 mice was ATF6, which could regulate the expression of five proteins (Fig. 3C): Calr, Hsp90b1, Pck1 (spot 1), Acox1 (spot 3), and Cpt2 (carnitine *O*‐palmitoyltransferase 2, mitochondrial; spot 6; an enzyme involved in acyl transfer across the mitochondrial inner membrane for β‐oxidation in the matrix) (Fig. 3A,B, Table 2, and Table S2 [Sheet B]). Although the upregulation of Pck1/Acox1/Cpt2 was common between liver and kidney, the two major organs for both gluconeogenesis and β‐oxidation (Figs. 2C and 3C), the downregulation of Calr and Hsp90b1 was rather kidney‐specific, which may place ATF6 upstream of PPARα in IPA analysis (Table S2 [Sheet B]). ATF6 is an endoplasmic reticulum stress‐regulated transmembrane transcriptional factor that is activated by its proteolytic cleavage with site 1 and site 2 proteases; the resultant cytosolic portion translocates to the nucleus, binds to ER stress response elements, and induces ER stress‐responsive genes 38. Recent evidence indicates that the interruption of hepatocellular autophagy attenuates the ATF6‐mediated unfolded protein response 39; thus, conversely, renal autophagy during fasting might induce ATF6 activation. In addition, KLF15, a member of the Krüppel‐like family of transcriptional factors, has been shown to regulate gluconeogenesis and KLF15‐deficient mice displayed severe hypoglycemia after overnight fasting 40; accordingly, 2‐day fasting induced KLF15 in two gluconeogenic organs, the liver (71st highest score) and the kidney (third highest score) (Table S2 [Sheet A and B]).

The other major finding of this study was TP53‐mediated transcriptional regulation in the thymus, spleen, and liver; TP53 was listed as the second, first, and fourth highest scoring upstream regulator, respectively (Figs. 4C and 5C; Table S2 [Sheet A, C, and D]). TP53 has been described as ‘the guardian of the genome’ because it regulates ‘thousands 41’ of target genes to prevent genome mutation and is encoded by the most frequently mutated gene in human cancer; however, TP53 also regulates multiple cellular responses including autophagy 42, inflammation, pluripotency, and energy metabolism 43, 44. A recent study mentioned that fasting robustly increases (stabilizes) TP53 in the mouse liver via hepatocyte autonomous and AMP‐activated protein kinase‐dependent posttranscriptional mechanisms, thereby regulating gluconeogenesis and amino acid catabolism 8. In addition, TP53‐deleted mice became hypoglycemic and showed defective utilization of hepatic amino acids upon fasting 8. Of note, TP53 regulated somewhat different sets of genes in the thymus and spleen. Two‐day fasting upregulated five proteins (Krt8/18 [keratin, type II cytoskeletal 8/18], Anxa4 [annexin A4], Actb [actin, cytoplasmic 1], Serpinb6 [serpin B6], and Gsn [gelsolin]), and downregulated five proteins (Hsp90ab1 [heat shock protein HSP 90‐beta], Actn1 [alpha‐actinin‐1], Hspd1 [60 kDa heat shock protein, mitochondrial], Hspa8 [heat shock cognate 71 kDa protein], Rpsa [40S ribosomal protein SA], and Ldha [l‐lactate dehydrogenase A chain]) in the thymus (Fig. 4C, Table 3, and Table S1 [Sheet C]). In contrast, the fasting upregulated five proteins (Apoa1 [apolipoprotein A‐I], Krt8, Gstm5 [= Gstm1, glutathione *S*‐transferase Mu 1], Ada [adenosine deaminase], and Actn1/4 [mixtures of alpha‐actinin 1/4], and downregulated six proteins (Hsp90ab/aa1 [mixtures of heat shock protein HSP 90‐beta/alpha], Stat 1 [signal transducer and activator of transcription 1], Ezr [ezrin], Rpsa, Krt18, and Aco2 [aconitate hydratase, mitochondrial] in the spleen (Fig. 5C, Table 4, and Table S1 [Sheet D]). In the spleen, nearly half of the proteins with altered expression during fasting were TP53 target gene proteins, which may place TP53 at the top of the upstream regulator lists (Table S2 [Sheet D]). Furthermore, TP53 regulation of 12 proteins (among 19 proteins identified) was liver‐specific (Table S2 [Sheet A]). Fasting/calorie restriction has been also shown to reduce age‐related diseases including cancer 45; and therefore, fasting‐induced TP53 regulation could be involved in such systemic tumor suppression.

MYCN and HTT were listed as the first and third highest scoring upstream regulators in the thymus (Fig. 4C), where NFKBIA and RARB were as the second and third highest scoring upstream regulators in the spleen (Fig. 5C). However, they only regulated only 6, 7, 5, and 3 proteins, respectively, in the IPA analysis, and their physiological roles await further investigations. In conclusion, this proteomic study revealed protein remodeling in response to fasting in the mouse liver, kidney, thymus, and spleen that could be transcriptionally regulated by PPARα and/or TP53. These findings could open new perspectives to understating the systemic effects of single fasting in animal experiments.

## Author contributions

SK, JY, and II conceived and designed the project; SK, JY, HO, YT, SY, and NA acquired the data; SK, JY, HO, YT, SY, NA, and II analyzed and interpreted the data; SK and II wrote the manuscript.

## Supporting information


**Table S1.** (A) Liver: Differentially expressed proteins upon 2‐day fasting identified by 2D DIGE and MALDI‐TOF/MS analyses. (B) Kidney: Differentially expressed proteins upon 2‐day fasting identified by 2D DIGE and MALDI‐TOF/MS analyses. (C) Thymus: Differentially expressed proteins upon 2‐day fasting identified by 2D DIGE and MALDI‐TOF/MS analyses. (D) Spleen: Differentially expressed proteins upon 2‐day fasting identified by 2D DIGE and MALDI‐TOF/MS analyses.

**Table S2.** (A) Liver: Upstream regulator analysis of differentially expressed proteins upon 2‐day fasting by Ingenuity Pathway Analysis (IPA). (B) Kidney: Upstream regulator analysis of differentially expressed proteins upon 2‐day fasting by Ingenuity Pathway Analysis (IPA). (C) Thymus: Upstream regulator analysis of differentially expressed proteins upon 2‐day fasting by Ingenuity Pathway Analysis (IPA). (D) Spleen: Upstream regulator analysis of differentially expressed proteins upon 2‐day fasting by Ingenuity Pathway Analysis (IPA).
